# Luteogenesis and Embryo Implantation Are Enhanced by Exogenous hCG in Goats Subjected to an Out-of-Season Fixed-Time Artificial Insemination Protocol

**DOI:** 10.3390/biology10050429

**Published:** 2021-05-12

**Authors:** Jorge A. Bustamante-Andrade, César A. Meza-Herrera, Rafael Rodríguez-Martínez, Zurisaday Santos-Jimenez, Oscar Ángel-García, Leticia R. Gaytán-Alemán, Ulises N. Gutierrez-Guzman, Amaury Esquivel-Romo, Francisco G. Véliz-Deras

**Affiliations:** 1 Unidad Laguna, Universidad Autónoma Agraria Antonio Narro, Periférico Raúl López Sánchez y Carretera A Santa Fe, Torreón 27054, Coahuila, Mexico; abaj_86@hotmail.com (J.A.B.-A.); rafael.rdz.mtz@gmail.com (R.R.-M.); mvz_zusan@hotmail.com (Z.S.-J.); mvz.oscar_2207@hotmail.com (O.Á.-G.); zukygay_7@hotmail.com (L.R.G.-A.); velizderas@yahoo.com (F.G.V.-D.); 2 Facultad de Agricultura y Zootecnia, Universidad Juárez del Estado de Durango, Venecia 35111, Durango, Mexico; 3 Unidad Regional Universitaria de Zonas Áridas, Universidad Autónoma Chapingo, Bermejillo 35230, Durango, Mexico; ulisesnoelg@yahoo.com.mx (U.N.G.-G.); amauryer@hotmail.com (A.E.-R.)

**Keywords:** goats, reproduction, seasonality, FTAI, reproductive efficiency, embryo implantation

## Abstract

**Simple Summary:**

In temperate and subtropical ecosystems, goats are classified as seasonal-polyestrous, generating a seasonality in their products (i.e., milk, meat). In order to abolish or reduce this reproductive seasonality, exogenous hormones, such as human chorionic gonadotropin (hCG), have been used to stimulate the development of ovarian follicles, triggering ovulation, corpus luteum formation, while increasing serum progesterone levels, fetal growth, prolificacy, and kidding rate. We tested the possible effect of two doses of hCG (i.e., 100 vs. 300 IU) in the improvement of the luteal function and embryo implantation in anovulatory goats subjected to an estrus induction protocol, and then, to a fixed-time artificial insemination protocol (FTAI). The highest values for fecundity rate, corpus luteum area, as well as embryonic efficiency index 1, were favored in goats treated with 300 IU hCG 14 days post-artificial fertilization. The embryonic efficiency and the index embryonic implantation efficiency were favored in goats treated with 300 IU hCG 7 and 14 days post-artificial insemination. This work contributes to explore the most suitable use of exogenous hormones that favor the out-of-season reproductive outcomes in goats and to boost the embryo implantation efficiency.

**Abstract:**

The aim of this study was to evaluate the possible effect of two doses of hCG (100 and 300 IU) applied at two different times (7 and 14 d) after a fixed-time artificial insemination protocol (FTAI) upon some variables involved in the embryonic implantation rate in goats during the natural deep anestrous season (April, 25° north). The experimental units considered crossbred, multiparous, anovulatory goats (n = 69, Alpine, Saanen, Nubian x Criollo), with average body weight (43.6 ± 5.7 kg) and body condition score (1.86 ± 0.28 units) located in northern–semiarid Mexico (25° N, 103° W). Once the goat’s anestrus status was confirmed, goats were subjected to an estrus induction protocol. Upon estrus induction confirmation, goats (n = 61) were subjected to a FTAI procedure. Immediately after the FTAI, the goats were randomly distributed to five experimental groups: (1). G100-7 (n = 13) 100 IU, hCG 7 d post-FTAI, (2). G100-14 (n = 12) 100 IU hCG, 14 d post-FTAI, (3). G300-7 (n = 12) 300 IU, hCG, 7 d post-FTAI, (4). G300-14 (n = 12) 300 IU hCG 14 d post-FTAI, and (5). Control group, CONT (n = 12) 0.5 mL saline, 7 and 14 d post-FTAI. The response variables conception rate (39.36 ± 0.23), fertility rate (27.96%), prolificacy rate (1.1 ± 0.29 kids), ovulation rate (0.74 ± 0.20 corpus luteum) corpus luteum diameter (10.15 ± 0.59 mm), embryo number (1.58 ± 0.20), and embryo implantation rate (48.96%), did not differ between treatments. However, while the variables fecundity rate (67%), embryo efficiency index-1 (33.99 ± 0.20%), and embryo efficiency index-2 (27.94 ± 0.30%) were favored by the G300-14 treatment, the corpus luteum area was favored (*p* < 0.05) by both G300-7 (113.30 ± 0.19 mm^2^) and G300-14 (103.04 ± 0.17 mm^2^). Such reproductive strategy emerges as an interesting approach, not only to enhance the out-of-season reproductive outcomes, but also to boost one of the main rulers defining the global reproductive efficiency of a heard, namely, the embryo implantation efficiency.

## 1. Introduction

Goat production is an important activity that has benefited human development since the dawn of our civilization; a significant percentage of the rural population in marginal ecosystems, from an ecological and social perspective, depends on this activity for the sustainability of their livelihoods [1,2]. Mexico has an inventory close to 9.0 million goats, highlighting The Comarca Lagunera (TCL), an agroecological region located in the arid north of Mexico, with respect to the levels of production and goat census at the national level with about 400,000 goats [3]; TCL ranks first in goat milk production in Mexico [2,4,5]. In recent years, a reduction in the goat inventory has occurred in TCL, although accompanied by increases in milk production [5]. This positive trend in the efficiency of dairy production suggests a greater selection pressure, both in replacement females, and in the selection of sires, with high genetic merit for dairy production, mainly Saanen and Alpine [2,5].

In this regard, both in temperate and subtropical ecosystems, goats are classified as seasonal-polyestrous; this seasonal reproduction in turn generates a seasonality in goat products such as meat, milk and their derivatives [6,7,8]. In TCL, the multiracial local goats show a period of deep anestrus from March to May; the reproductive season begins in August, ending in February [8,9,10,11]. In order to abolish or reduce this reproductive seasonality, exogenous hormones, such as human chorionic gonadotropin (hCG) with different doses (i.e., 50, 100, and 300 IU), have been used in goats. For instance, at the onset of the breeding season (i.e., 0, 50, 100, and 300 IU; [11]), 100 IU using different administration routes [12], as well as during the seasonal anestrous [13], and 100 IU either during the deep anestrous, and the transition from anestrous to the breeding season [14]. The administration of hCG in anestrus goats stimulates the development of ovarian follicles, triggering ovulation, and the formation of the corpus luteum [11,15]. In the same way, hCG has been used to increase not only plasma progesterone levels, but prolificacy, fetal growth, and kidding percentage, by using 150 and 300 IU at the onset of the breeding season [16]. The critical period of gestation is directly linked to the maternal recognition of pregnancy process (MRPP), which initiates because of the presence of the conceptus in the uterus; it activates an antiluteolytic mechanism designed to protect the corpus luteum [17,18]. Certainly, by safeguarding luteogenesis, the synthesis and release of progesterone, necessary to initiate embryo implantation and establish gestation, is throughout the MRPP; the main antiluteolytic signal released by conceptus–trophoblast cells is the interferon tau (INFτ) [17,18,19,20].

In goats, gestation is maintained by progesterone secreted by the corpus luteum [21]; any luteal dysfunction generating low progesterone concentrations during the early stages of pregnancy will seriously compromise the embryo implantation process [22,23]. Indeed, about 20% of embryonic mortality attributed to luteal failure is concentrated during the 15 days after the ovum fertilization process [22,24], particularly during the period of conceptus elongation, which occurs prior to the MRPP, ending with embryo implantation [23]. Certainly, an inadequate luteal function is one of the most relevant causes of pregnancy failure in goats [25,26]. Building on the above findings, we tested the hypothesis that a low administration of hCG after fixed-time artificial insemination (FTAI), positively affects the MRPP in goats during the anestrous period, improving luteogenesis and embryonic implantation rate, in anovulatory goats subjected to an estrus induction protocol, and subsequently exposed to FTAI, managed under extensive conditions. Moreover, a positive effect of hCG upon other reproductive response variables should be expected; our study aims to elucidate such inquiries.

## 2. Materials and Methods

### 2.1. General

All of the experimental procedures, methods, and handling of the experimental test units used in this study complied with the guidelines for the ethical use, care, and well-being of research animals at the international [27] and national [28] levels, with institutional approval reference number UAAAN-UL-18-3059.

### 2.2. Location, and Environmental Conditions

The study was carried out in the Ejido Venecia, Gómez Palacio, Durango, Mexico (25° 47′ NL, 103° 21′ WL, altitude = 1111 m). Rainfall occurs from June to September with an annual average of 266 mm (range 163 to 440 mm). This region has a dry climate with an average annual temperature of 21 °C varying from 37 °C (May–August) to 0 °C in winter [29]. The goats were fed under the extensive sedentary grazing system predominant in the Comarca Lagunera, consuming the native vegetation, such as Buffelgrass (*Cenchrus ciliare*), Bermuda grass (*Cynodon dactylon*), Navajita (*Bouteloua* spp.), Johnson (*Sorghum halepense*), Chamizo (*Atriplex canescens*), as well as Mesquite sprouts and fruits (*Prosopis glandulosa*), Huizache (*Acacia farnesiana*), shrubs, and eventually crop residues, such as sorghum, melon, cotton watermelon, forage oats [30]. The goats went out to grazing at 10:00 a.m. and returned to the corral at 06:00 p.m. The goats were hand-milked once a day at 07:00 a.m. All goats were subcutaneously dewormed (Ivermectin 1%, Baymec, Bayer, Mexico City, Mexico) and also received doses of vitamin A (500,000 IU), D3 (75,000 IU), E (50 mg) (Vigantol: ADE + Selenium, 250 mL, Zapopan, Jalisco, Mexico), one-month prior the onset of the study; water, shades, and mineral salts (17% P, 3% Mg, 5% Ca, and 75% NaCL) were freely available. All of the goats received daily individual nutritional supplementation (10:00 a.m.) during 10 d pre- and 10 d post-FTAI; the supplement consisted of 100 g of rolled corn (8.6% CP) and 500 g of alfalfa hay (18% CP), as suggested by [31].

### 2.3. Animals and Their Management

#### 2.3.1. Female Goats: Confirmation of the Anovulatory Status, Estrus Induction Protocol, and Estrus Detection with Aproned Males

From a commercial crossbred goat herd (n = 155; Alpine, Saanen, Nubian x Criollo), a total of 69 multiparous, anovulatory goats, with 43.6 ± 5.7 kg live weight, 1.86 ± 129 0.28 units body condition score, and 2–4 kidding were selected and identified with earrings for their best management during the experimental period. Anovulation was determined in March by means of an ultrasound fitted to a 7.5 MHz transrectal transducer (Aloka SSD-500, Richmond, Canada). Each goat underwent an ultrasound on days 14 and 7 prior to the application of progesterone (P4), in order to confirm the absence of corpora lutea in both ovaries. Once anovulation was confirmed, the goats were subjected to an estrus induction protocol. On day -1, all females received 20 mg of P4 (Progesvit^®^, Brovel, i.m., Irapuato, Guanajuato, Mexico) in order to avoid the presence of short cycles. The following day, day zero (0 d), the goats received 200 IU of hCG (Chorulon^®^, Intervet, Mexico City, Mexico.) i.m., in order to stimulate the formation of the antrum in the ovarian follicles in advanced stages of development, and promote, in turn, ovulation. Both P4 and hCG were applied around 08:00 h of the respective day. The detection of estrus considered the use of three sexually active adult males provided with aprons to prevent them from copulating with the female. The goat was considered in standing estrus when it remained immobile when the male rode it. Immediately, the female was removed from the pen for the male to continue detecting heat. From the initial 69 goats exposed to the estrus induction protocol, only 61 were declared in estrus based on their interaction with the aproned males.

#### 2.3.2. Male Goats: Setting Bucks for Semen Extraction and Semen Quality Assessment

Male goats (n = 3; Granadina breed; 2.5 years old) were used; this breed shows an optimal level of adaptability and productivity in free-ranging production systems [32]. Besides, this breed displays a reduced reproductive seasonality; females are considered as continuous polyestrous, presenting kidding all-year-around without the need for the use of exogenous hormones [33,34]. Besides, bucks have a lessened period of sexual rest, nonetheless, in our study, in order to induce an intense sexual activity and libido, the bucks used for semen collection, received an application of 50 mg of testosterone (T) (Testosterone-50, steroidal androgenic, i.m., Lab Brovel, Mexico City, Mexico) every third day for three weeks’ prior to the onset of the trial as previously suggested in this geographical area [35]. Males received a daily nutritional supplementation 60 d prior semen collection, based on 2 kg alfalfa hay per male (2.3 Mcal/kg, 18% PC/kg DM) plus 600 g of a commercial concentrate (1.7 Mcal/kg, 14% PC/kg DM) as previously recommended [31] in order to enhance both metabolic and physiological outputs [36]. By the end of April, the semen was extracted with the use of an artificial vagina (Walmur, Montevideo, Uruguay); the ejaculate was evaluated macro and microscopically, considering motility, viability, and sperm concentration. For the FTAI process, only ejaculates with the following characteristics were used: volume of ≥0.5 mL, sperm mass motility of ≥3 (scale 0–5), sperm concentration ≥2500 × 10^6^ cells/mL, and progressive motility ≥70%. Once the semen quality of the three bucks was confirmed, in order to avoid any confounded sire effect upon the embryo implantation rate, the collected samples of semen were mixed, to obtain a final composite semen sample. Thereafter, the semen was diluted (Optidyl^TM^, Cryo-Vet, Leon Guanajuato, Mexico) to obtain a final concentration of 200 × 10^6^ per dose (0.2 mL) and was kept in plastic straws in refrigeration (4 °C); prior to the FTAI, another microscopic analysis was implemented to verify the quality of the diluted semen.

### 2.4. Fixed-Time Artificial Insemination (FTAI)

Immediately after the estrus induction protocol (i.e., 20 mg P4 and 200 IU hCG), and once the estrus status was confirmed, all goats (n = 61), in late April, were exposed to the FTAI protocol. Briefly, once the sperm quality was re-evaluated, the diluted semen was transferred to a container with water at 30 °C. The FTAI procedure was carried out in the handling pen, using a vaginoscope for veterinary use (Walmur-Veterinary Instrument, Montevideo, Uruguay) equipped with a light source. The semen was deposited in the pericervical area 58 h after the application of 200 UI of hCG, then, the next day at 09:00 h (72 h after the application of 200 UI hCG), a second FTAI was carried out; that is, all females were subjected to the FTAI protocol twice; 58 and 72 h after applying the 200 IU of hCG.

### 2.5. Conformation of the Experimental Groups: The Use of hCG Post-FTAI to Enhance the Embryo Implantation Rate

**Figure 1:**
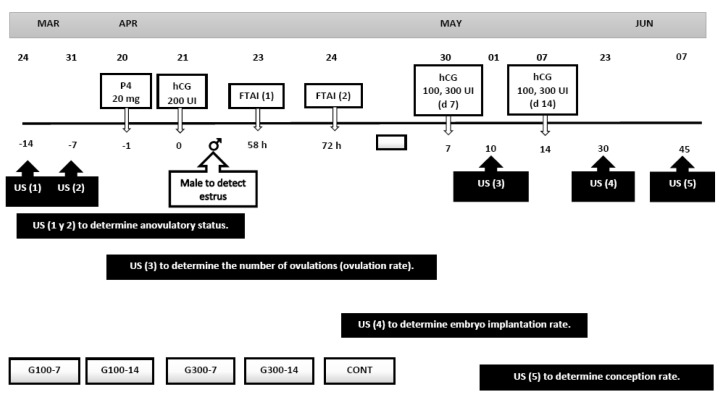
Schematic representation of the experimental protocol. The application of hCG (100 and 300 UI) was on day 7 and 14 post-FTAI. Ultrasound (US) was performed to determine anovulatory status, the number of ovulations (ovulation rate), the embryo implantation rate, and conception rate.

Once all the goats were artificially inseminated (FTAI, n = 61), they were randomly distributed to five experimental treatments considering two doses of hCG (100 and 300 IU, Chorulon^®^, Intervet, Mexico City, Mexico) and two application times (7 and 14 d post-FTAI), plus the Control group. Therefore, the experimental groups were conformed as follows: (1). G100-7 (n = 13) 100 IU, hCG 7 d post-FTAI, (2). G100-14 (n = 12) 100 IU hCG, 14 d post-FTAI, (3). G300-7 (n = 12) 300 IU, hCG, 7 d post-FTAI, (4). G300-14 (n = 12) 300 IU hCG 14 d post-FTAI, and (5). Control group, CONT (n = 12) 0.5 mL saline 7 and 14 d post-FTAI. In all the experimental groups, the hCG was applied i.m. between 08:00 and 09:00 on the neck, either 7 or 14 days post-FTAI. Therefore, the possible effect of these five experimental treatments upon embryo implantation rate, luteogenesis as well as other reproductive variables, was evaluated (Figure 1).

### 2.6. Response Variables

#### 2.6.1. Body Weight, Body Condition Score, and Estrus Induction

Body weight (BW) and body condition score (BCS) were recorded at the beginning of the experimental period and when performing the corresponding ultrasounds. To determine BW, an electronic scale with a capacity of 250 kg and an accuracy of 50 g was used (Torrey 110v/220v, Digital Industrial Scale, Jalisco, Mexico). Moreover, BCS was determined by one experienced technician as previously described [37], considering a scale from 1 (very thin) to 4 (very fat).

#### 2.6.2. Ovulation Percentage, Ovulatory Rate, and Luteogenesis

The percentage of ovulating females was determined on days 0 and 10 after the estrus induction protocol by means of a transrectal ultrasonography evaluation (Aloka SSD-500, Richmond, Canada) using a 7.5 MHz linear probe. The ovulatory rate was determined through ultrasonography on day 10 after the FTAI; the luteal area was determined considering the diameter of the corpora lutea observed in the ultrasonography.

#### 2.6.3. Embryo Implantation Rate, and Other Reproductive Variables

On d-30 post-FTAI, the embryo implantation rate was determined through transrectal ultrasonography using color Doppler equipment (Chison ECO-5, with 12 inch probe); at this time, the embryonic implantation process should have occurred in the uterus. Other reproductive response variables collected within treatment were conception rate: determined on day 45 post-FTAI, considering the pregnant goats/inseminated goats, fecundity rate: considering the number of fetuses per inseminated goat, fertility rate: the number of pregnant females that gave birth, prolificacy rate: determined at parturition and considering the number of kids born per pregnant female. Moreover, two indices were developed in order to ponder the success of the embryo implantation rate with respect to both conception rate and fecundity rate: Embryo Efficiency Index 1: (embryo implantation rate) (conception rate/100), and Embryo Efficiency Index 2: (embryo implantation rate) (fecundity rate/100).

### 2.7. Statistical Analyses

A first linear model was developed to evaluate the possible relationship of hCG dose (i.e., 100 vs. 300) and period of administration (i.e., 7 vs. 14 d post-FTAI) with respect to body weight (BW, kg), corpus luteum diameter (CLD, mm), corpus luteum area (CLA, mm^2^). Regarding percentage and counts variables body condition score (BCS, units), estrus induction (EI, %), conception rate (CR, %), fertility rate (FR, %) prolificacy rate (PR, %), fecundity rate (FC, %), ovulation rate (OR, units), embryo number (EN, units), embryo implantation rate (EIR, %), embryo efficiency index-1(EEI-1, %) and embryo efficiency index-2 (EEI-2, %), since they do not fit normal distribution, they were log^10^ transformed prior to ANOVA to overcome skewness. Least-squares means and standard errors for each experimental treatment were computed; multiple mean comparisons were performed through the Fisher’s LSD–LSMEANS option of the PROC GLM of SAS. Since all the experimental treatments were individually evaluated, each goat within the experimental group was defined as the experimental unit; treatment differences were accepted if *p* < 0.05. All the analyses were computed through the procedures of SAS (SAS Inst. Inc. version 9.4, Cary, NC, USA).

## 3. Results

### 3.1. Body Weight, Body Condition Score, and Estrus Induction

No differences (*p* > 0.05) regarding body weight (BW, 45.6 ± 1.21 kg), body condition score (BCS, 1.88 ± 0.08 units), or estrus induction (EI, 88.41% 61/69) occurred among experimental groups (Table 1). Prior to the estrus induction protocol, the ultrasound scanning confirmed that 69/69 female goats were anestrous previous to the FTAI protocol.

### 3.2. Conception Rate, Fertility Rate, Prolificacy Rate, and Fecundity Rate

As observed in Table 2, no differences (*p* > 0.05) regarding conception rate, fertility rate, and prolificacy rate occurred across experimental groups when considering two doses of hCG (100 and 300 IU) applied at two different periods of time (7 and 14 d), along with the Control group. Respective averages for the response variables were (39.36 ± 0.23%, 27.96%, 1.1 ± 0.29 kids). Interestingly, however, the phenotypic expression of fecundity rate differed (*p* < 0.05) among the experimental groups, favoring the G300-14 goats.

### 3.3. Ovulation Rate, Corpus Luteum Area and Diameter, Embryo Number, Embryo Implantation Rate, and Embryo Efficiency Indexes

No differences (*p* > 0.05) among treatments occurred for the response variables ovulation rate (0.74 ± 0.20 units), corpus luteum diameter (10.15 ± 0.59 mm), embryo number (1.58 ± 0.20), and embryo implantation rate (48.96%). In contrast, the largest corpus luteum area was favored (*p* < 0.05) by the G300 group, irrespective of the time of application, regarding the G100-7, G100-14, and CONT, with corresponding values of 113.3, 103.0 vs. 72.7, 79.3, and 45.9 mm (Table 3). Moreover, while no differences (*p* > 0.05) occurred among the G100-7, G100-14, G300-7, and CONT treatment groups neither for EEI1 nor EEI2, the largest EEI1 was favored (*p* < 0.05) by the G300-14 group while the EE2 was favored by both the G300-7 and G300 14 (*p* < 0.05).

## 4. Discussion

The results obtained in this study do not support our working hypothesis, which proposed that a low dose of hCG (i.e., 100 vs. 300 IU) would be effective to improve luteal function and embryo implantation in anovulatory goats subjected to an estrous induction protocol and, subsequently, to a fixed-time artificial insemination (FTAI) protocol. Indeed, the highest values for the response variables: fecundity rate (FC), corpus luteum area (CLA), embryonic efficiency index 1 (EEI1) was favored by the G300-14 group, while the embryonic efficiency index 2 (EEI2) was favored by both the G300-7 and G300 14 groups. That is, a high dose of hCG administered 14 d after the FTAI, improved the embryonic implantation rate. In this regard, hCG not only has a structure equivalent to LH, with 70% similarity, and a longer half-life (i.e., 39 h vs. 6 h), it also has 30% similarity with FSH, which favors ovarian stimulation at the level of theca cells and FSH receptors in granulosa cells, while both positively influence the critical period of early pregnancy; the embryo implantation process [38]. The present study contributes to a better understanding regarding the use of exogenous hormones that enhance the reproductive performance of previously anovulatory females, being an interesting alternative strategy to reduce reproductive seasonality and improve the embryo implantation rate.

At the beginning of the study, the state of anestrus was confirmed in 100% of the goats (69/69), subsequently, 88.41% (61/69) of them responded positively to the estrus induction protocol. Such trend coincides with other studies evaluating the use of 20 mg of P4 and diverse doses of hCG in both intensive and extensive production systems, in Alpine goats and Crossbred goats, as well as in the deep anestrous (April) as in the time of transition to estrus (June), concluding that this protocol was able to break the seasonal anestrus in more than 90% of goats [11,13,15,16]. Another interesting aspect in our study is that both BW and BCS did not differ among experimental groups in April, with similar values to those reported in goats managed under an extensive sedentary system in northern Mexico [39]. They also reported that after the dry season (i.e., winter) the BW of goats is significantly decreased under these marginal production systems, generating embryonic or fetal losses in the early stages of gestation.

Regarding the conception rate, fertility rate, and prolificacy rate, they did not differ among experimental groups; the obtained values were higher than those formerly reported [40]. A myriad of environmental, genetic, metabolic, endocrine, physiological, sanitary, and even tissue-related factors, among others, which can be involved in the differences observed among studies. Moreover, all the experimental groups exhibited embryonic losses, so that fertility and the prolificacy rate decreased, coinciding with a previous study [39]. Nonetheless, in our study, the fecundity rate was higher in the G300-14 group, that is, a greater number of fetuses was observed with respect to the number of inseminated goats in that group, coinciding with a study which evaluated the use of different doses of GnRH and hCG four days post-insemination in Merino ewes, in relation to fertility and pregnancy rate [41]. Concerning gestational losses, an incidence of 70% of abortions has been reported in crossbred goats under extensive systems in the arid north of Mexico, affecting the global prolificacy of the herds [25]. In goats, the corpus luteum is the only source of progesterone production since the placenta does not produce it during pregnancy [42]; P4, in turn, influences the synthesis and action of both interferon-tau (INFτ) and prostaglandins, as well as the expression of oxytocin receptors and certain genes activated by INFτ [18,24,43]. This physiological scenario can generate changes in the critical levels of certain hormones and growth factors that might compromise the correct progression of maternal recognition of pregnancy, conceptus survival, and embryo implantation [17,24,44]. In our study, the administration of two doses of hCG on days 7 and 14 did not show favorable results in some response variables. However, in other studies [11,16,30] using different hCG doses in small ruminants, higher levels of plasma progesterone were observed, in addition to better performance in other physiological parameters. Therefore, in future research, it would be interesting to correlate reproductive response variables, such as ovulation rate, percentage of ovulation, diameter of the corpus luteum, and luteal area with respect to plasma progesterone levels. Regarding the variables ovulatory rate, corpus luteum diameter, number of embryos, and embryo implantation rate, they did not differ among treatments. In this respect, it is important to consider that both hCG doses could have achieved the minimum required threshold in order to promote a similar reproductive response between treatments. Such results are in line with previous studies evaluating the use of hCG in seasonal anestrus goats, observing a similar effect of hCG on ovulatory rate [11,13]. The core idea, or central aim of this study was to evaluate the possible similar action of 100 or 300 IU of hCG upon some reproductive out-of-season outcomes in female goats under marginal production systems. A possible explanation of such lack of effect in the 100 IU-groups could be related to the extremely high sensitivity of the hypothalamic–pituitary axis to the negative retroaction of the gonadal E2 experimented by goats that were facing a deep anestrus. Most hormonal actions depend upon certain thresholds in order to provoke a defined response. So, a low hCG dose, a deep anestrous phase, added to a marginal production system may have conspired against better reproductive upshots. Another key issue is that the greater the size of the evaluated population, the largest the statistical robustness of the obtained results. Certainly, while non-larger enough replicate number tend to mask interesting results regarding the use of exogenous hormones to enhance out-of-season reproductive outcomes, the measurement of different metabolic and reproductive hormones, as well as diverse blood analytes should increase the possibility to better understand the diverse interactions occurring at physiological level while affecting the observed outcomes. Such issues must be clearly considered in future studies.

Embryo implantation is a very important and complex process which depends on an intricate while sophisticated crosstalk among the ovary, the uterus, and the conceptus; this complex process is fundamental since the highest rate of gestational losses occurs during the embryo pre-implantation period [8]. The increase in the levels of both INFτ [23,45] and P4 [23,46] augments the likelihoods of a successful embryo implantation in ruminants. In our study, no differences occurred regarding the EIR between hCG doses, similar to that observed in other studies evaluating the use of hCG upon embryo implantation [22]; yet, the administration of hCG enhanced other reproductive variables with respect to the use of GnRH and the control group [47]. Interestingly, in our study, the largest luteal area was observed in the G300-14 group, agreeing with a previous study evaluating 300 IU of hCG 7d post-estrus in Toggenburg goats under an intensive system (21° South), observing a positive relationship between luteal area and hCG dose [48]. Such a physiological scenario also coincides with other studies using different hCG doses in small ruminants under different production systems (i.e., extensive vs. intensive), across different years/seasons, and diverse latitudes, concluding that the use of hCG was able to induce estrous activity [11,15,16,49,50].

Undoubtedly, the greater the size of the evaluated population, the largest the statistical robustness of the obtained results. Certainly, while non-larger, enough replicate numbers tend to mask interesting results regarding the use of exogenous hormones to enhance out-of-season reproductive outcomes, the measurement of different metabolic and reproductive hormones, as well as diverse blood analytes should increase the possibility to better understand the diverse interactions occurring at the physiological level while affecting the observed outcomes. Such issues must be carefully considered in future studies.

The maternal recognition of pregnancy is a process by which the conceptus indicates its presence to the mother in order to prolong the life of the corpus luteum, the P4 release while enhancing the pregnancy rate [17,24]. In order to ponder the success of the embryo implantation rate, with respect to the fertility and fecundity rate, two embryo implantation efficiency indices were constructed in our study: EEI1 and EEI2. The values generated for EEI1 favored the G300-14 group, while the observed EEI2 values favored the G300-7 and G300-14 groups, heightening the maternal recognition of pregnancy. This scenario suggests that the G300-14 had increased levels in either INFτ or in the receptors to the said molecule, at either the endometrial or ovarian level, enhancing the antiluteolytic mechanism destined to preserve the progestational state indispensable for the success of embryo implantation. In ruminants, the undeniably antiluteolytic signal is INFτ produced by trophoblast cells [24,42]. Taking into account both the time of application with respect to the FTAI and the half-life of hCG (i.e., about 40 h), in the G300-14 group, the embryo implantation rate could had been favored by enhancing the action of INFτ, which under normal conditions, it is mostly released on days 16 and 18 after the fertilization process. One of the main actions of INFτ is to prevent the pulsatile release of prostaglandin F2a (PGF2a) whose primary objective is to promote luteolysis or regression of the corpus luteum [17,51]. Therefore, the positive effects of exogenous hCG on goat embryonic development cannot be ruled out [52], and would explain the possible physiological scenario exerted by the G300-14 experimental group.

The precise site of action of hCG upon its positive effects regarding the embryo implantation success cannot be established without further studies. At the endometrial level, and considering a genomic approach, INFτ not only induces or activates various INF-stimulated genes (*ISGs*), but also positively regulates various genes closely related to the conceptus elongation process, its implantation, and the establishment of pregnancy [51,53]. Interestingly, the *IFNT* genes have only been described in ruminants within the genus Artiodactyla (i.e., cattle, sheep, and goats), emerging from an ancestral gene 36 million years ago, and over time, surely through rearrangements, and/or indel events that combined specific arrangements in such an ancestral gene (i.e., INF-ω) with a trophoblast-specifying promoter–enhancer domain [54,55]. While there is a low INFτ expression in the blastocyst stage, a massive upregulation occurs during the initial stages of the conceptus elongation; the INFτ expression vanishes with the attachment of the trophectoderm to the uterine endometrium. The main promoter element that controls expression is an ETS-2/AP-1 enhancer element [55]. Growth factors and cytokines released by the maternal endometrium have been implicated in the control of *IFNT* gene transcription through the activation of ETS-2 [54]. This timely expression of *IFNT* is not only necessary to rescue the corpus luteum to secure the gestation process, but can also be an indicator of the suitability of the conceptus, thus acting as a critical factor dictating the continuation of gestation in goats and other ruminants. Moreover, *IFNT* acts through STST1/STA2-independent paths to uphold the activation of *ISGs*, supporting not only the P4 release by the small and large luteal cells, but also enhancing the transport of glucose and amino acids essential for the growth and development of the conceptus [18]. Much remains to be revealed; therefore, it is essential to endure with the assessment of novel, but innovative, reproductive and nutritional strategies that allow avoiding embryonic losses during the embryo implantation process, which, together with the free life period of the fertilized ovum, are critical to transit towards optimal reproductive efficiency outcomes of the female goat.

## 5. Conclusions

The response variables—conception rate, fertility rate, ovulation rate, corpus luteum diameter, embryo number, prolificacy rate, and embryo implantation rate—did not differ among experimental groups. Nonetheless, the 300 IU dose of hCG administered 14 d after the fixed-time artificial insemination protocol improved fecundity rate, corpus luteum area, as well as the embryonic efficiency index 1, while enhanced embryonic efficiency index 2 values were favored by both the G300-7 and G300 14. EEI1 and EEI2 were developed in order to ponder the success of the embryo implantation rate with respect to both the conception and fecundity rates; once such outcomes were weighed, the G300-14 group displayed the best embryonic implantation efficiency values. The present study contributes to attaining a better understanding, regarding the use of exogenous hormones that favor the reproductive performance of previously anestrus females. Consequently, such a reproductive strategy emerges as an interesting approach, not only to enhance the out-of-season reproductive responses, but also to boost one of the main rulers defining the global reproductive efficiency of a heard, namely, the embryo implantation rate. These results are of physiological importance and reproductive significance to the goat industry, and may embrace potential translational applications.

## Figures and Tables

**Table 1:** Least-square means ± standard error for body weight (BW), body condition score (BCS), and estrus induction (EI) according to the experimental treatment to be exposed after to the fixed time artificial insemination protocol, in multiracial, multiparous, and anovulatory goats (n = 61, Alpine, Saanen, Nubian x Criollo) managed under extensive conditions in Northern Mexico (April, 25° N) ^1^.

Variables	G100-7 (n = 13)	G100-14 (n = 12)	G300-7 (n = 12)	G300-14 (n = 12)	CONT (n = 12)	*p* Value
BW, (kg)	45.5 ± 0.80	45.8 ± 1.2	45.9 ± 0.82	45.2 ± 1.50	45.6 ± 1.71	0.99
BCS, (units)	1.9 ± 0.09	2.0 0.07	1.8 ± 0.07	1.9 ± 0.11	1.8 ± 0.07	0.62
EI, (n, %)	13/14 (92.8)	12/15 (80.0)	12/13 (92.3)	12/13 (92.3)	(12/14) 85.71	0.64

^1^ No differences (*p* > 0.05) for any variable occurred among experimental groups.

**Table 2:** Least-square means ± standard error for conception rate (CR), fertility rate (FR) prolificacy rate (PR), and fecundity rate (FC) according to the experimental treatment considering two doses of hCG (100 and 300 IU) and applied at two different times (7 and 14 d), and the Control group (CONT) after a fixed time artificial insemination protocol in multiracial, multiparous, and anovulatory goats (n = 61, Alpine, Saanen, Nubian x Criollo) managed under extensive conditions in Northern Mexico (April, 25° N).

Variables	G100-7 (n = 13)	G100-14 (n = 12)	G300-7 (n = 12)	G300-14 (n = 12)	CONT (n = 12)	*p* Value
CR (n, %)	5/13 (38.5 ± 0.15)	5/12 (41.7 ± 0.28)	3/12 (25 ± 0.22)	7/12 (58.3 ± 0.20)	4/12 (33.3± 0.30)	0.54
FR (n, %)	3/13 (23.1)	3/12 (25)	3/12 (25)	6/12 (50)	2/12 (16.7)	0.71
PR (n)	1.7 ± 0.29	0.6 ± 0.33	1.3 ± 0.33	1.4 ± 0.21	0.5 ± 0.29	0.53
FC (n, %)	5/13 (38) ^b^	2/12 (17) ^b^	4/12 (33) ^b^	8/12 (67) ^a^	2/12 (17) ^b^	0.06

^a,b^ Response variables with different superscripts within lines, differ (*p* > 0.05).

**Table 3:** Least-square means ± standard error for ovulation rate (OVR), corpus luteum diameter (CLD), corpus luteum area (CLA), embryo number (EN), embryo implantation rate (EIR), embryo efficiency index-1(EEI-1) and embryo efficiency index-2 (EEI-2), according to the experimental treatment considering two doses of hCG (100 and 300 IU), and applied at two different times (7 and 14 d), and the Control group (CONT) after a fixed time artificial insemination protocol in multiracial, multiparous, and anovulatory goats (n = 61, Alpine, Saanen, Nubian x Criollo) managed under extensive conditions in Northern Mexico (April, 25° N).

Variables	G100-7 (n = 13)	G100-14 (n = 12)	G300-7 (n = 12)	G300-14 (n = 12)	CONT (n = 12)	*p* Value
OVR *n*	0.61 ± 0.17	0.75 ± 0.22	0.58 ± 0.19	1.27 ± 0.17	0.58 ± 0.23	0.3
CLD (mm)	9.54 ± 0.48	9.91 ± 0.77	11.88 ± 0.71	11.22 ± 0.47	8.21 ± 0.51	0.10
CLA (mm^2^)	72.73 ± 0.18 ^b^	79.37 ± 0.22 ^b^	113.30 ± 0.19 ^a^	103.04 ± 0.17 ^a^	45.96 ± 0.19 ^b^	0.02
EN *n*	1.75 ± 0.13 ^a^	1.40 ± 0.24	1.67 ± 0.21	1.57 ± 0.20	1.50 ± 0.24	0.78
EIR *n* (%)	8/13 (61.5)	5/12 (41.7)	6/12 (50)	7/12 (58.3)	4/12 (33.3)	0.61
EEI1 (%) ^1^	23.68 ± 0.12 ^b^	17.39 ± 0.24 ^b^	12.5 ± 0.21 ^b^	33.99 ± 0.20 ^a^	11.09 ± 0.24 ^b^	0.02
EEI2 (%) ^2^	19.00 ± 0.37 ^b^	9.91 ± 0.20 ^b^	20.30 ± 0.25 ^a^	27.94 ± 0.30 ^a^	5.66 ± 0.18 ^b^	0.02

Different letters between columns show difference (*p* > 0.05). Data are presented as mean ± standard error of the mean.^1^ Embryo Efficiency Index 1 = (implantation rate) (conception rate/100). ^2^ Embryo Efficiency Index 2 = (implantation rate) (fecundity rate/100).

## Data Availability

Not applicable.
